# Triglyceride-to-HDL Cholesterol Ratio Is Associated with Ischemic Stroke Risk in Patients—With Paroxysmal Atrial Fibrillation

**DOI:** 10.3390/metabo16020110

**Published:** 2026-02-03

**Authors:** Ciprian Ilie Rosca, Daniel Florin Lighezan, Doina Georgescu, Horia Silviu Branea, Nilima Rajpal Kundnani, Ariana Violeta Nicoras, Romina Georgiana Bita, Daniel Dumitru Nisulescu

**Affiliations:** 1 Department of Internal Medicine I—Medical Semiotics I, Centre for Advanced Research in Cardiovascular Pathology and Haemostasis, “Victor Babes” University of Medicine and Pharmacy, Eftimie Murgu Sq. No. 2, 300041 Timisoara, Romaniadlighezan@umft.ro (D.F.L.); doina.georgescu@umft.ro (D.G.); 2 Department of Internal Medicine I—Medical Semiotics II, “Victor Babes” University of Medicine and Pharmacy, Eftimie Murgu Sq. No. 2, 300041 Timisoara, Romania; 3 Department of Cardiology—Ambulatory Internal Medicine, “Victor Babes” University of Medicine and Pharmacy, Eftimie Murgu Sq. No. 2, 300041 Timisoara, Romania; 4 Research Centre of Timisoara, Institute of Cardiovascular Diseases, “Victor Babes” University of Medicine and Pharmacy, 300041 Timisoara, Romania; 5 Doctoral School, “Victor Babes” University of Medicine and Pharmacy, Eftimie Murgu Sq. No. 2, 300041 Timisoara, Romaniadaniel.nisulescu@umft.ro (D.D.N.); 6 2nd Department, Radiology and Medical Imaging, General and Dento-Maxillary Imaging, Dental Medicine Faculty, “Victor Babes” University of Medicine and Pharmacy, 300041 Timisoara, Romania

**Keywords:** atrial fibrillation type, paroxysmal atrial fibrillation, TG/HDLc ratio, ischemic stroke

## Abstract

**Background:** Ischemic stroke remains the most feared complication of atrial fibrillation (AF), and thromboembolic risk is commonly estimated using clinical scores that may not fully capture the cardiometabolic dimension of cerebrovascular vulnerability. The aim of this research was to assess whether additional parameters can be used, to predict ischemic stroke risk in patients with AF, in order to explore whether TG/HDL-C may complement conventional clinical risk scores for ischemic stroke risk stratification in PAF, and to better characterize a metabolically high-risk phenotype beyond the recommendations provided by the CHA_2_DS_2_-VA score, which is useful but still far from perfect in predicting AF-associated ischemic stroke risk. **Methods:** In this retrospective, single-center observational study, we evaluated whether the triglyceride-to-high-density lipoprotein cholesterol ratio (TG/HDLc), a simple surrogate of atherogenic dyslipidemia and insulin resistance, is associated with ischemic stroke risk in patients with paroxysmal atrial fibrillation (PAF). We screened 1111 consecutive AF admissions between 1 January 2015 and 31 December 2016 and, from these 1111 AF cases, we extracted only the patients with PAF for analysis. Patients were stratified based on TG/HDLc values into two groups, Group 1 (TG/HDLc > 2.5; n = 155) and Group 2 (TG/HDLc < 2.5; n = 194). Statistical analysis was performed with MedCalc v23.4.0 using Chi-square and unpaired/Welch’s t-tests as appropriate, Pearson correlations, Kaplan–Meier analysis with log-rank testing, Cox regression for first ischemic stroke, and multivariable logistic regression to identify independent correlates of TG/HDLc > 2.5. **Results:** Patients with TG/HDLc > 2.5 had a significantly higher prevalence of ischemic stroke after AF onset compared with those with TG/HDLc < 2.5 (37.4% vs. 21.1%, *p* = 0.0008), despite similar CHA_2_DS_2_-VA and HAS-BLED scores, and also exhibited a higher burden of cerebrovascular and neurodegenerative findings, including cortical atrophy and cerebral lacunarism. Ischemic stroke-free survival curves diverged significantly over time (log-rank *p* = 0.0186), and an elevated TG/HDLc ratio was associated with a 68% higher hazard of first ischemic stroke (HR 1.68; 95% CI 1.09–2.60). In multivariable analysis, type 2 diabetes mellitus (OR 4.53), hyperuricemia (OR 3.83), dyslipidemia (OR 1.94), stroke (OR 1.77), and cortical atrophy (OR 4.48) were independently associated with TG/HDLc > 2.5. **Conclusions:** These findings suggest that TG/HDLc identifies a metabolically high-risk PAF phenotype associated with greater cerebrovascular burden and reduced ischemic stroke-free survival, providing an inexpensive and broadly available marker that may complement conventional clinical risk scores.

## 1. Introduction

Atrial fibrillation (AF), the most common cardiac arrhythmia, has increased in prevalence in the last 20 years, despite all the efforts made to decrease its prevalence [1,2].

Ischemic stroke related to atrial fibrillation remains the most feared complication in real-life patients and may occur even in the presence of an appropriate treatment [3].

Despite anticoagulation strategies and structured risk assessment, thromboembolic events continue to occur, suggesting that conventional clinical scores may not fully capture stroke susceptibility in all AF phenotypes. Stroke risk stratification is mainly based on the CHA_2_DS_2_-VA score; however, this approach is largely driven by clinical comorbidities and does not explicitly incorporate markers of cardiometabolic risk, atherosclerotic burden, or insulin-resistance–related dyslipidemia. This may be particularly relevant in paroxysmal AF (PAF), where thromboembolic risk can still be substantial and may be underestimated in some patients [4].

High-density lipoprotein cholesterol (HDLc), considered the “good” cholesterol, appears to exert different effects in women and men: low levels are associated with an increased risk of AF in women but not in men [5], while levels > 60 mg/dL are associated with protection against AF [6]. The protective effect of HDLc on AF has also been reported. HDLc levels were directly and positively correlated with AF incidence [7] and even with new-onset AF [8].

Low HDLc levels were not found to be directly associated with increased AF incidence, similarly to elevated triglyceride (TG) levels and low-density lipoprotein cholesterol (LDLc) levels [9].

Metabolic syndrome (MetSy) represents a clustering of cardiovascular risk factors linked through shared pathophysiological mechanisms [10,11,12]. The presence of dyslipidemia has been associated with higher inflammatory markers and oxidative stress markers, as well as with endothelial dysfunction in young patients without overt cardiovascular disease but who were overweight or obese compared with those of normal weight [13].

The presence of metabolic syndrome predisposes to a threefold increase in the risk of ischemic stroke [10,13].

Elevated serum TG levels are associated with increased intravascular fibrin deposits and intravascular thrombosis, as well as with the triggering of oxidative stress and inflammation, which may become chronic with persistent hypertriglyceridemia over time [14]. Additionally, higher TG levels are associated with increased insulin resistance, whereas subsequent reductions in TGs are associated with improvement in insulin resistance [15]. Reduced peripheral tissue responsiveness to insulin has been termed insulin resistance and has been demonstrated to be an important pathophysiological factor in the development and progression of cardiovascular diseases [16].

Plasma TG levels are among the main important determinants of small, dense LDLc, which has been shown to play an important role in increasing cardiovascular risk [17].

On the other hand, low TG values and high LDLc values have been associated with an increased risk of deep vein thrombosis and venous thromboembolism [18]

The use of non-traditional lipid components may provide more accurate information regarding the cardiovascular and metabolic risk associated with dyslipidemias [19,20,21].

Higher TG values and a higher TG/HDLc ratio have been associated with increased ischemic stroke incidence, even in patients with normal BMI, but also is associated with atherosclerotic progression, with a similar pattern observed for low HDLc values [19]. Other studies have failed to demonstrate a predictive role of TG/HDLc for major adverse cardiovascular events [20].

The TG/HDL-C is an inexpensive and widely available marker linked to atherogenic dyslipidemia and insulin resistance, and has been associated with incident ischemic stroke and vascular disease in population studies. However, whether TG/HDL-C can help identify PAF patients at higher ischemic stroke risk beyond clinical scores remains insufficiently defined [21].

The aim of this study was to explore whether TG/HDL-C may complement conventional clinical risk scores for ischemic stroke risk stratification in PAF, and to better characterize a metabolically high-risk phenotype.

## 2. Material and Methods

This was a retrospective, single-center observational study including consecutive hospital admissions with a diagnosis of AF between 1 January 2015 and 31 December 2016. The unit of analysis was the patient. For patients with multiple admissions, the first eligible admission during the study window was considered the index record; subsequent admissions were used only to quantify hospitalization burden. Before starting this study, we obtained approvals from the Hospital management and the Ethics Committee of the “Victor Babes” University of Medicine and Pharmacy, Timișoara.

Inclusion criteria: (i) age ≥ 18 years; (ii) paroxysmal AF diagnosed according to ESC definitions/guidelines; (iii) availability of a lipid panel allowing calculation of TG/HDL-C at the index admission; and (iv) available documentation to ascertain ischemic stroke events after AF onset.

Exclusion criteria: (i) persistent/permanent AF; (ii) valvular AF (moderate–severe mitral stenosis or mechanical heart valve); (iii) missing lipid data (TG or HDL-C) preventing TG/HDL-C calculation; and (iv) missing outcome ascertainment data.

**Figure 1:**
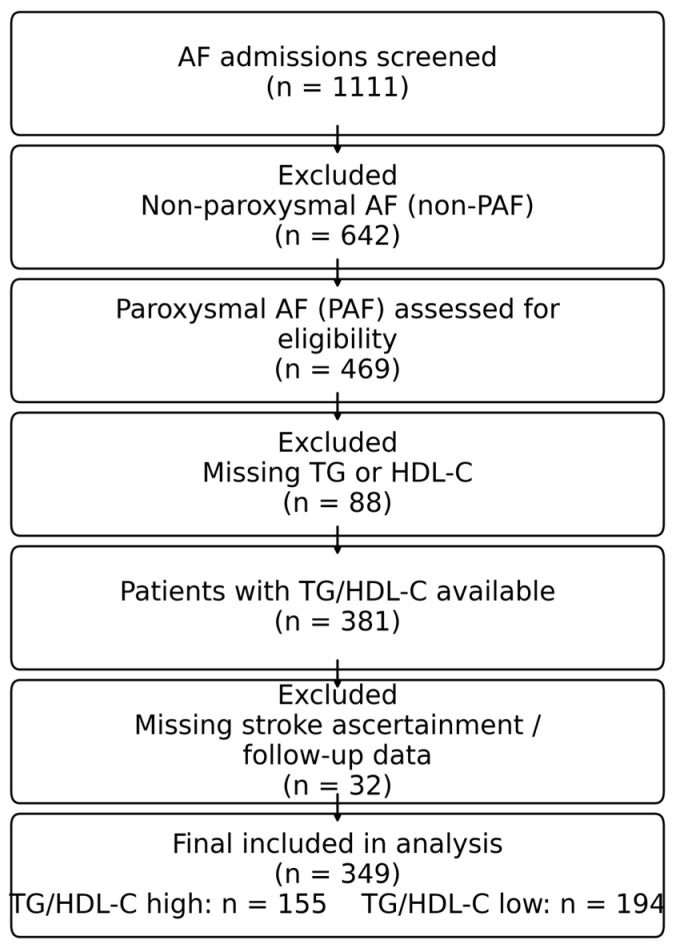
Flow diagram of patient selection and exclusions.

During the period, a total of 1111 AF cases were recorded, only 349 patients were eligible (Figure 1). TG/HDL-C was calculated from the lipid panel obtained at the index hospital admission. Ischemic stroke events were extracted from specialist documentation as occurring at any time after AF onset, including events documented before the index admission as well as events recorded during subsequent follow-up. Therefore, in this retrospective analysis, TG/HDL-C should be interpreted primarily as a marker of an underlying, long-term cardiometabolic phenotype associated with cumulative cerebrovascular burden and recurrence propensity, rather than as a strictly prospective predictor of stroke events that may have preceded lipid assessment. The presence of ischemic stroke, dementia, and PD was noted if the diagnosis was made by a neurologist, psychiatrist, or radiologist (in the present admission or previous medical evaluation). After patient identification, we divided these patients into two groups. The first is the group with a value of TG/HDLc ratio above 2.5, and is a group with 155 patients (Group 1, n = 155, TG/HDLc ratio ≥ 2.5), and the second one with TG/HDLc ratio under 2.5, a group with 194 patients (Group 2, n = 194, <2.5). We pre-specified TG/HDL-C > 2.5 as a “high-ratio” category based on prior cardiovascular literature using this threshold to define higher cardiometabolic/atherogenic risk. In sensitivity analyses, TG/HDL-C was evaluated as a continuous variable and by quartiles to ensure robustness. The blood samples were processed in the Hospital’s Central Clinical Laboratory, using the same machine and technique. Blood samples for lipid assessment were collected from peripheral venous blood at the index admission, typically in the morning as part of routine evaluation, after an overnight fast (≥8 h) whenever feasible. Triglycerides and HDL-C were measured in the hospital central laboratory using a standardized assay platform. TG/HDL-C was calculated from a single measurement obtained at the index admission (no repeated lipid measurements were available for longitudinal averaging).

AF was defined according to the European Society of Cardiology guidelines on the management of atrial fibrillation [22].

Ischemic stroke was defined as an acute focal neurological deficit consistent with cerebral infarction, diagnosed by a neurologist and supported by available neuroimaging and/or hospital discharge documentation. Hemorrhagic strokes were excluded when imaging or clinical documentation indicated bleeding. Because imaging was performed as part of routine care rather than a standardized research protocol, detailed stroke subtyping and severity grading were not consistently available.

Metabolic syndrome (MetSy) was defined according to the International Diabetes Federation (IDF) criteria. The diagnoses is based on the presence of central obesity (assessed by waist circumference) or obesity, together with any two of the following: serum TGs ≥ 150 mg/dL (or treatment for hypertriglyceridemia), reduced HDL cholesterol ≤ 40 mg/dL in men and ≤50 mg/dL in women, elevated systolic blood pressure ≥ 130 mmHg or diastolic blood pressure ≥ 85 mmHg, and raised fasting plasma glucose (RFPG) ≥ 100 mg/dL or a prior diagnosis of type 2 diabetes (T2DM) [10]. Although TG/HDLc is not included among the classical definitions of MetSy [11] it is of crucial importance, as it represents a surrogate marker of peripheral insulin resistance that can be easily calculated at no additional cost. Its components are part of the routine assessment of cardiovascular patients, and both components of the ratio, when considered individually, are included in the classical MetSy definition.

Body mass index (BMI) was calculated as weight (kg)/height^2^ (m^2^) using measurements recorded at the index admission. BMI categories followed WHO definitions: overweight ≥ 25 kg/m^2^ and obesity ≥ 30 kg/m^2^. Statistical analysis was performed using MedCalc Statistical Software v23.4.0 (MedCalc Software Ltd., Ostend, Belgium) with a significant *p* < 0.05. Continuous variables were summarized as mean ± standard deviation (SD) (and 95% confidence intervals where appropriate) and/or median (interquartile range, IQR) depending on distribution; categorical variables were expressed as counts (percentages). Between-group comparisons were performed using: unpaired student’s *t*-test for normally distributed continuous variables with homogeneous variances, or Welch’s t-test when variances were unequal; Chi-square test for categorical variables, with Fisher’s exact test used when expected cell counts were small; Pearson correlation to assess linear associations between TG/HDLc and continuous metabolic parameters. Given the large number of univariable comparisons, these analyses were considered exploratory and hypothesis-generating; therefore, no formal correction for multiplicity was applied. Marginal *p*-values should be interpreted cautiously, with greater emphasis placed on effect sizes and confidence intervals. Effect sizes for categorical associations were reported as odds ratios (OR) and risk ratios (RR) with 95% confidence intervals. Multivariable logistic regression was used to identify independent variables associated with membership in the high TG/HDLc group (TG/HDLc ≥ 2.5), as a phenotype-characterization analysis. This model was intended to describe clinical and neurological features clustering within the high TG/HDLc phenotype and should not be interpreted as implying a causal direction; stroke risk prediction was assessed separately using Kaplan–Meier/Cox analyses for first ischemic stroke. Kaplan–Meier curves and the log-rank test were used for ischemic stroke-free survival comparisons, and Cox proportional hazards regression was applied to estimate hazard ratios (HR) for first ischemic stroke.

## 3. Results

From a demographic perspective, the number of patients with an elevated TG/HDLc ratio (Group 1, n = 155) was comparable to that of patients with a low TG/HDLc ratio (Group 2, n = 194). The rural/urban distribution did not differ significantly between groups (39.4% vs. 43.8% from rural areas, *p* = 0.4020), and the sex distribution was also similar; the proportion of men was 52.9% in Group 1 compared with 44.8% in Group 2 (*p* = 0.1472), with no statistically significant differences (Table 1).

Regarding comorbidities and risk factors, the profile of patients with an elevated TG/HDLc ratio was clearly more unfavorable. Group 1 had a significantly higher proportion of patients with multiple hospital admissions (23.9% vs. 15.5%, *p* = 0.0479), suggesting a greater overall disease burden. Hydrostatic varicose veins were more frequently observed in Group 1 compared with Group 2 (19.4% vs. 10.3%, *p* = 0.0167), as was obesity, present in 41.9% of patients with elevated TG/HDLc vs. 18.6% in Group 2 (*p* = 0.0356). Type 2 diabetes mellitus (T2DM) was more than twice as prevalent in Group 1 (51.6% vs. 19.1%, *p* = 0.0007), and MetSy was also significantly more common (38.1% vs. 14.9%, *p* = 0.0125). Dyslipidemia (Table 2) was almost universal among patients with an elevated TG/HDLc ratio (96.1% vs. 53.1%, *p* = 0.0322). Hyperuricemia without signs of tophaceous disease (Table 2) was also more frequent in this group, at approximately 14.9% compared with 7.1% in Group 2 (*p* = 0.0223). No significant differences were observed for overweight (Table 2), defined as BMI < 30 kg/m^2^, chronic obstructive pulmonary disease (COPD), bronchial asthma, hypothyroidism, or anxiety–depressive disorders (Table 3 and Table 4).

Major cardiovascular conditions were highly prevalent in both groups (Table 5). Overall, heart failure (HF) was present in more than two-thirds of patients, with no significant difference between Group 1 and Group 2 (74.8% vs. 72.2%, *p* = 0.5751). Both heart failure with preserved ejection fraction (HFpEF) and heart failure with reduced ejection fraction (HFrEF) showed similar prevalence across the two groups (HFpEF 23.9% vs. 25.8%, *p* = 0.6836; HFrEF 51.0% vs. 46.4%, *p* = 0.3960). Arterial hypertension was extremely common in both groups, without significant differences (85.2% vs. 86.6%, *p* = 0.7015), and carotid atheromatosis had a comparable distribution (23.2% vs. 19.1%, *p* = 0.3438). The only major cardiovascular difference observed was the presence of chronic coronary syndrome, which was more frequent in Group 1 than in Group 2 (64.5% vs. 53.6%, *p* = 0.0402).

Cerebrovascular and neurological conditions were significantly more frequent among patients with an elevated TG/HDLc ratio (Patients in Group 1 had a higher prevalence of ischemic stroke occurring after AF onset compared with Group 2 (37.4% vs. 21.1%, *p* = 0.0008). In addition, cortical atrophy was more than five times more common in Group 1 (11.6% vs. 2.1%, *p* = 0.00003), and cerebral lacunarism occurred in 9.7% of patients with elevated TG/HDLc vs. 4.1% in Group 2 (*p* = 0.0380). Parkinson’s disease was also significantly more prevalent among patients with a higher TG/HDLc ratio (5.8% vs. 1.0%, *p* = 0.0113). Cognitive decline was numerically more frequent in Group 1 (7.7% vs. 4.6%), but did not reach statistical significance (*p* = 0.2265). These neurological and neuroimaging findings were extracted from routine clinical documentation and were not assessed using a standardized protocol; therefore, they should be interpreted as associative and hypothesis-generating rather than causal.

Patients in Group 1 had a higher prevalence of ischemic stroke occurring after AF onset compared with Group 2 (37.4% vs. 21.1%, *p* = 0.0008) (Table 6). In addition, cortical atrophy was more than five times more common in Group 1 (11.6% vs. 2.1%, *p* = 0.00003), and cerebral lacunarism occurred in 9.7% of patients with elevated TG/HDLc vs. 4.1% in Group 2 (*p* = 0.0380). Parkinson’s disease was also significantly more prevalent among patients with a higher TG/HDLc ratio (5.8% vs. 1.0%, *p* = 0.0113). Cognitive decline was numerically more frequent in Group 1 (7.7% vs. 4.6%), but did not reach statistical significance (*p* = 0.2265). These neurological and neuroimaging findings were extracted from routine clinical documentation and were not assessed using a standardized protocol; therefore, they should be interpreted as associative and hypothesis-generating rather than causal.

These neurological and neuroimaging findings were extracted from routine clinical documentation and were not assessed using a standardized protocol; therefore, they should be interpreted as associative and hypothesis-generating rather than causal.

Regarding treatment, therapeutic patterns differed mainly for medications related to heart failure and the metabolic profile (Table 7). No significant differences were observed between the two groups in the use of vitamin K antagonist anticoagulants, beta-blockers, angiotensin receptor blockers (ARBs), or statins. However, patients in Group 1 received spironolactone more frequently (63.9% vs. 53.1%, *p* = 0.0329) and also had a significantly higher use of furosemide compared with Group 2 (*p* = 0.0430), consistent with more severe congestion and a greater need for diuresis. Propafenone was prescribed more often in patients with an elevated TG/HDLc ratio (10.3% vs. 4.5%, *p* = 0.0444), suggesting a greater need for rhythm control. Proton pump inhibitors were used more frequently in Group 1 (50.5% vs. 39.4%, *p* = 0.0403), and metformin was also administered much more often to patients with elevated TG/HDLc (14.8% vs. 4.1%, *p* = 0.0292), in line with the high prevalence of type 2 diabetes mellitus in this group. No relevant differences were observed in alprazolam use. Overall, the medication profile reflects a more complex cardiometabolic and neurological burden in patients with TG/HDLc > 2.5.

Quantitative parameters and risk scores are summarized in Table 8, Table 9 and Table 10. Group 1 (TG/HDLc > 2.5) was older than Group 2, whereas renal function (eGFR) and electrolyte values were comparable between groups (Table 8). Thromboembolic and bleeding risk scores (CHA_2_DS_2_-VA and HAS-BLED) did not differ significantly; however, Group 1 had a higher documented ischemic stroke count (Table 9). Laboratory testing showed a more atherogenic and metabolically adverse profile in Group 1, including higher triglycerides, LDL-C and total cholesterol, higher uric acid and glycaemia, and lower HDL-C (Table 10). All analyses were performed using Welch’s t-test and the unpaired t-test, depending on variance homogeneity.

Table 11 summarizes univariable associations between categorical variables and TG/HDL-C category. Overall, the high TG/HDL-C group clustered with an unfavorable cardiometabolic profile—particularly obesity, type 2 diabetes, metabolic syndrome, dyslipidemia, and hyperuricemia—supporting the interpretation of TG/HDL-C as a marker of a higher-risk metabolic phenotype. In addition, markers of greater clinical complexity (e.g., multiple hospital admissions) and venous disease were more frequent in the high TG/HDL-C group. The strongest associations were observed for cerebrovascular and neurological conditions, including a history of ischemic stroke and neuroimaging-related findings such as cortical atrophy and lacunar infarcts, as well as neurodegenerative diagnoses. Conversely, the low TG/HDL-C group showed reciprocal (inverse) associations for the same variables. Detailed effect sizes (OR/RR) and confidence intervals are provided in Table 11. 

The correlation analysis (Table 12) between the TG/HDLc ratio and metabolic markers identified a significant association only with blood glucose. The TG/HDLc ratio showed a weak but statistically significant positive correlation with glucose levels (r = 0.17, *p* = 0.0013), with a 95% confidence interval for the correlation coefficient ranging from 0.067 to 0.271. This suggests that as the TG/HDLc ratio increases, glucose values also tend to be higher, which is consistent with the close relationship between an atherogenic lipid profile and disturbances in glucose metabolism. In contrast, no significant correlations were found between TG/HDLc and LDLc (r = 0.06, *p* = 0.2869) or between TG/HDLc and uric acid (r = 0.04, *p* = 0.4490), with the 95% confidence intervals including zero. These results suggest that, in this cohort of patients with paroxysmal atrial fibrillation, the TG/HDLc ratio may reflect glycometabolic dysregulation rather than showing a linear association with LDLc or hyperuricemia, despite the fact that the latter are more frequent in the group with elevated TG/HDLc. Correlations were assessed using Pearson’s correlation coefficient.

### Stroke Risk Analyses (Time-to-Event Models)

**Figure 2:**
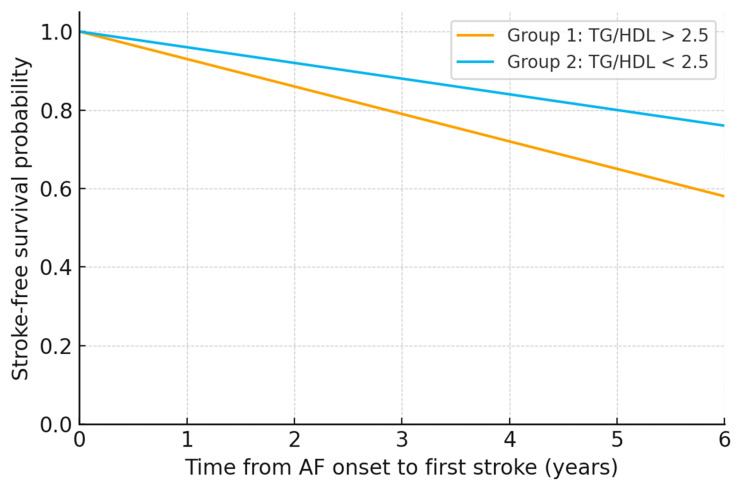
Kaplan–Meier curves for time from atrial fibrillation onset to first documented ischemic stroke, stratified by TG/HDL-C category measured at the index admission. Because TG/HDL-C was obtained at index admission, the curves should be interpreted as reflecting association with a cardiometabolic phenotype linked to cumulative ischemic stroke burden after AF onset, rather than a purely prospective prediction for events occurring before lipid assessment. Secondary analysis: phenotype clustering (logistic regression).

Ischemic stroke-free survival according to TG/HDLc ratio. The prognostic impact of an elevated TG/HDLc ratio on cerebrovascular events was further explored using Kaplan–Meier analysis (Figure 2). During follow-up, a first ischemic stroke occurred in 37.4% of patients with TG/HDLc > 2.5, compared with 21.1% of those with TG/HDLc < 2.5. Ischemic stroke-free survival curves progressively diverged over time, and the difference between groups was statistically significant (log-rank χ^2^ = 5.54, *p* = 0.0186). In the corresponding Cox regression model, patients in Group 1 (TG/HDLc > 2.5) had a 68% higher hazard of a first ischemic stroke compared with those in Group 2 (TG/HDLc < 2.5), with a hazard ratio of 1.68 (95% C.I. 1.09–2.60), consistent with the greater burden of cardiometabolic and neurological comorbidities described above.

Secondary phenotype characterization (not causal direction): To characterize the clinical clustering associated with an elevated TG/HDLc ratio, we fitted a multivariable logistic regression model with TG/HDLc group membership as the dependent variable (Table 13). A history of ischemic stroke was independently associated with membership in Group 1 (TG/HDLc > 2.5) (OR 1.77, 95% C.I. 1.07–2.92, *p* = 0.0258). The presence of cortical atrophy showed a strong independent association with Group 1 (OR 4.48, 95% C.I. 1.42–14.14, *p* = 0.0105), suggesting that patients with structural brain lesions tend to cluster within the high-risk metabolic phenotype defined by TG/HDLc > 2.5. This phenotype model complements the time-to-event analyses and is not intended to establish causal direction (Table 13).

Among cardiometabolic factors, type 2 diabetes mellitus was a very strong independent predictor, increasing the odds of having an elevated TG/HDLc ratio by more than fourfold (OR 4.53, 95% C.I. 2.81–7.29, *p* < 0.0001). Dyslipidemia remained significantly associated with Group 1 even after adjustment (OR 1.94, 95% C.I. 1.25–4.43, *p* < 0.0001). Hyperuricemia also retained a robust independent effect (OR 3.83, 95% C.I. 1.84–7.95, *p* = 0.0003), placing uric acid within the same cardiometabolic risk cluster together with diabetes and dyslipidemia.

## 4. Discussion

Cardiovascular risk management should begin at an early age and continue throughout life. Increasing awareness of cardiovascular risk and pursuing an effective, sustained approach to its components should be a shared goal of both healthcare professionals and patients.

Reframing healthcare systems by placing the individual at the center of decisions regarding their own health must become a universal priority. Along with recognizing the major role individuals play in managing their own health, there should also be accountability mechanisms that encourage adherence to maintaining health and preventing diseases that may affect them.

Interventions targeting the components of dyslipidemia should be initiated before the clinical manifestations of atherosclerosis occur [17].

Emerging evidence regarding medications that modulate insulin resistance including effects on lipid metabolism provides additional support for the importance of addressing dyslipidemias with the aim of bringing serum lipid levels back within normal ranges [15].

The use of novel markers, or readily available surrogates of established markers that do not require additional investment in equipment, could be extremely useful in cardiovascular disease management and may help reduce the cost of caring for cardiac patients [23,24,25].

Our data suggest an association between an atherogenic lipid profile, defined by an elevated TG/HDLc ratio, and the presence of coronary artery disease, and further indicate that a higher TG/HDLc ratio correlates not only with vascular risk factors but also with a greater burden of cerebrovascular and neurodegenerative lesions.

The principal finding of the present study is that a high TG-to-HDL cholesterol (TG/HDLc) ratio identifies a subgroup of patients with paroxysmal atrial fibrillation (PAF) who have a significantly higher burden of ischemic stroke, despite having comparable CHA_2_DS_2_-VA and HAS-BLED scores. Patients with TG/HDLc > 2.5 exhibited a higher prevalence of prior ischemic stroke, a greater number of cerebrovascular events per patient, and significantly reduced ischemic stroke-free survival. These findings suggest that TG/HDLc captures a cardiometabolic risk dimension that is insufficiently reflected by conventional clinical risk scores used in atrial fibrillation.

Our findings indicate that an elevated TG/HDL-C ratio is associated with a higher risk of incident ischemic stroke in patients with paroxysmal atrial fibrillation (PAF). Given the retrospective observational design, these results should be interpreted as correlative rather than causal. TG/HDL-C likely captures an integrated cardiometabolic risk phenotype that may increase vascular vulnerability and contribute to both atherosclerotic and small-vessel pathways, coexisting with AF-related thromboembolism rather than acting as an isolated causal driver.

TG/HDLc ratio as an integrative cardiometabolic marker: The TG/HDLc ratio has emerged as a robust surrogate of atherogenic dyslipidemia and insulin resistance, two interconnected pathophysiological mechanisms central to cardiovascular and cerebrovascular disease [20,26,27,28]. Elevated TG levels reflect increased hepatic very-low-density lipoprotein (VLDL) production and impaired lipolysis, while reduced HDL cholesterol is associated with dysfunctional reverse cholesterol transport, altered apolipoprotein composition, and loss of HDL’s anti-inflammatory and antioxidant properties [13,16,26]. Together, these alterations signal a systemic shift toward a pro-atherogenic, pro-inflammatory, and prothrombotic metabolic milieu. In our cohort, patients with elevated TG/HDLc demonstrated a clear clustering of adverse metabolic traits, including obesity, type 2 diabetes mellitus, metabolic syndrome, hyperuricemia, and higher fasting glucose levels. These associations reinforce the concept that TG/HDLc reflects a global metabolic phenotype, rather than an isolated lipid abnormality. The strong independent association between type 2 diabetes mellitus and elevated TG/HDLc observed in multivariable analysis aligns with extensive evidence indicating that insulin resistance accelerates vascular aging, promotes endothelial dysfunction, and enhances thrombogenicity in patients with atrial fibrillation [15,16,28].

Metabolomic perspective on TG/HDLc ratio and vascular risk: from a metabolomic standpoint, TG/HDLc can be interpreted as a composite proxy of disturbed lipid flux, impaired lipoprotein remodeling, and altered energy substrate utilization. Recent metabolomic profiling studies have shown that elevated TG/HDLc correlates with increased circulating levels of branched-chain amino acids, acylcarnitines, ceramides, and other lipid intermediates strongly linked to insulin resistance and vascular inflammation [9,10,26]. These metabolic signatures promote mitochondrial dysfunction, oxidative stress, and impaired nitric oxide bioavailability, thereby facilitating endothelial injury and microvascular damage. Such metabolomic alterations offer a plausible mechanistic explanation for the increased cerebrovascular burden observed in our patients with elevated TG/HDLc. Rather than acting as a passive marker, TG/HDLc likely reflects an active metabolic state that promotes vascular vulnerability, particularly in the presence of atrial fibrillation.

Ischemic stroke risk beyond CHA_2_DS_2_-VA: A particularly relevant observation is that stroke prevalence, ischemic stroke burden, and stroke-free survival differed significantly between TG/HDLc groups despite similar CHA_2_DS_2_-VA scores. While CHA_2_DS_2_-VA remains the cornerstone of ischemic stroke risk stratification in AF, it does not incorporate parameters related to dyslipidemia, insulin resistance, or systemic inflammation. Several population-based studies have demonstrated that elevated TG/HDLc is associated with incident ischemic stroke, even among individuals with normal body mass index or without overt cardiometabolic disease [19,29,30]. Our findings extend these observations to patients with PAF, a subgroup in whom thromboembolic risk is often underestimated compared with persistent or permanent AF. This cohort included only patients with paroxysmal AF, so extrapolation to persistent or permanent AF should be cautious. The relationship between TG/HDL-C and ischemic stroke may differ across AF subtypes due to differences in AF burden, atrial remodeling, comorbidity clustering, and patterns of anticoagulation use. Future studies should evaluate whether TG/HDL-C performs similarly across AF phenotypes and whether AF burden modifies its predictive value. The observed hazard ratio of 1.68 for first ischemic stroke among patients with TG/HDLc > 2.5 underscores the potential prognostic value of this ratio beyond traditional clinical scoring systems. A key translational question is whether TG/HDL-C adds predictive information beyond conventional clinical scores. Although TG/HDL-C appears to capture a metabolically high-risk phenotype not fully reflected by CHA_2_DS_2_-VA, we did not perform formal reclassification metrics (e.g., NRI/IDI) in this dataset. Prospective validation should quantify whether adding TG/HDL-C to clinical scores improves discrimination and reclassification in a clinically meaningful manner. Cerebral microvascular injury and neurodegenerative associations: Beyond overt ischemic stroke, patients with elevated TG/HDLc showed a significantly higher prevalence of cortical atrophy, cerebral lacunar infarctions, and Parkinson’s disease. Associations between TG/HDL-C and imaging/neurological findings should be interpreted cautiously. These variables were extracted from routine clinical documentation and imaging reports when available, and neuroimaging was performed as clinically indicated rather than under a standardized research protocol. Consequently, these findings are exploratory and hypothesis-generating, vulnerable to detection bias and differences in healthcare utilization, and they do not imply causality or a uniform stroke mechanism. The particularly strong association with lacunar infarctions suggests a link between atherogenic metabolic profiles and cerebral small-vessel disease, which is increasingly recognized as metabolically driven rather than purely hypertensive or age-related [29,30]. Insulin resistance and dyslipidemia contribute to cerebral microangiopathy through endothelial dysfunction, chronic inflammation, impaired cerebral autoregulation, and blood–brain barrier disruption [13,16]. In parallel, altered lipid metabolism and mitochondrial dysfunction may promote neuroinflammation and neuronal vulnerability, providing a mechanistic framework for the observed association between elevated TG/HDLc and cortical atrophy. Given the known neuroprotective effects of functional HDL particles, reduced HDL quantity and quality may further exacerbate neurodegenerative processes over time.

High-risk plaques: studies using coronary CT angiography have shown that an elevated TG/HDLc ratio is independently associated with the presence of high-risk plaque features, as well as with an increased risk of major adverse cardiovascular events [31]. These observations may provide a direct mechanistic explanation for the increased ischemic stroke risk observed in our study, given that atheromatous plaques can develop at any arterial level, not only in the coronary circulation.

Metabolic phenotyping and clinical implications: these findings all together support the concept that TG/HDLc represents a metabolic fingerprint of cerebrovascular vulnerability in patients with paroxysmal atrial fibrillation. Rather than reflecting a single biochemical abnormality, this ratio integrates information across multiple metabolic pathways, including lipid transport, insulin signaling, oxidative stress, inflammation, and endothelial function. From a translational perspective, TG/HDLc is a simple, inexpensive, and widely available marker that may refine cerebrovascular risk assessment in AF patients, particularly those with intermediate CHA_2_DS_2_-VA scores. While our data do not justify modifying anticoagulation decisions solely based on TG/HDLc, they strongly support more aggressive cardiometabolic risk factor management, including weight control, glycemic optimization, and targeted lipid modulation, in patients with elevated ratios. This approach aligns with contemporary AF management strategies emphasizing integrated, holistic cardiovascular care [22]. TG/HDL-C may be most informative as a complementary marker in (i) patients with intermediate CHA_2_DS_2_-VA scores where residual metabolic risk may influence cerebrovascular vulnerability, (ii) younger PAF patients with low clinical scores but clear metabolic dysfunction, and (iii) patients with frequent healthcare utilization or multimorbidity where a “metabolic–vascular” phenotype is suspected. Importantly, our findings do not support altering anticoagulation decisions solely based on TG/HDL-C without prospective validation; rather, they support intensified cardiometabolic risk-factor management.

Limitations and future directions: The retrospective, single-center design limits causal inference and generalizability. Lipid parameters were assessed at a single time point, and longitudinal metabolic changes could not be evaluated. Residual confounding remains possible despite multivariable adjustment. A key limitation is temporality: TG/HDL-C was measured at index admission, whereas ischemic stroke ascertainment covered the period after AF onset and could include events occurring before lipid assessment. Therefore, TG/HDL-C is best viewed here as a marker of a chronic cardiometabolic phenotype associated with lifetime/cumulative cerebrovascular burden, and reverse causality cannot be fully excluded. Although we assessed multiple clinical and cardiometabolic characteristics, residual confounding is likely, including hypertension severity and control, smoking status, kidney disease stage, and medication exposure. These factors can influence both TG/HDL-C and ischemic stroke risk, and may not be fully captured in retrospective datasets. In particular, while oral anticoagulation use was recorded, detailed information on anticoagulation quality was not consistently available; therefore, confounding by anticoagulation quality cannot be excluded. Also, Although oral anticoagulation use was recorded, detailed data on anticoagulation quality were not available. Given that anticoagulation quality is a key determinant of stroke risk, residual confounding by treatment quality cannot be excluded. Future prospective multicenteric studies are warranted to validate these findings and to assess whether incorporating TG/HDLc into existing risk models improves ischemic stroke prediction in atrial fibrillation. Importantly, future research integrating targeted or untargeted metabolomic profiling may further elucidate the molecular pathways underlying the association between TG/HDLc and cerebrovascular risk. Such approaches could identify novel metabolic biomarkers and therapeutic targets, reinforcing the role of metabolic modulation as a complementary strategy in the prevention of AF-related cerebrovascular complications.

## 5. Conclusions

This study demonstrates that the TG-to-HDL cholesterol ratio serves as a metabolic signature of increased cerebrovascular vulnerability in patients with paroxysmal atrial fibrillation. Elevated TG/HDLc was associated with a higher prevalence of ischemic stroke, greater ischemic stroke burden, impaired ischemic stroke-free survival, and increased markers of cerebral small-vessel disease, despite comparable conventional thromboembolic risk scores.

By integrating information from lipid metabolism, insulin resistance, and vascular dysfunction, TG/HDLc appears to capture a high-risk metabolic endotype that is not reflected by standard clinical scoring systems. These results support the concept that metabolic dysregulation plays a critical role in atrial fibrillation–related cerebrovascular disease and highlight the potential value of incorporating metabolic biomarkers into risk stratification strategies. Future studies integrating metabolomic profiling may further elucidate the mechanistic pathways underlying this association and identify novel targets for preventive intervention.

Moreover, as a simple and widely available metric, TG/HDLc may be considered a potential adjunct marker to prompt comprehensive cardiometabolic risk assessment in PAF; however, prospective validation is required before any clinical implementation as a screening strategy.

## Figures and Tables

**Table 1:** Demographic characteristics of the two groups.

Demographic Characteristics	Group 1—TG/HDLc > 2.5 (n = 155)	Group 2—TG/HDLc < 2.5 (n = 194)	*p*-Value
N	155 (44.4%)	194 (55.6%)	
Rural/Urban	61/94 (39.4%/60.6%)	85/109 (43.8%/56.2%)	0.4020
Sex (M/F)	82/73 (52.9%/47.1%)	87/106 (45.1%/54.9%)	0.1472

N = number of patients, urban—cities, rural—villages, M—male, F—female, statistical test used—Chi Squared.

**Table 2:** Metabolical changes presented in both groups.

Metabolic Changes	Group 1—TG/HDLc > 2.5 (n = 155)	Group 2—TG/HDLc < 2.5 (n = 194)	*p*-Value
N	155 (44.4%)	194 (55.6%)	
Overweight	5/155 (3.2%)	12/194 (6.2%)	0.2025
Obesity	65/155 (41.9%)	36/194 (18.6%)	0.0356
T2DM	80/155 (51.6%)	37/194 (19.1%)	0.0007
Metabolic syndrome	59/155 (38.1%)	29/194 (14.9%)	0.0125
Dyslipidemia	149/155 (96.1%)	103/194 (53.1%)	0.0322
Hyperuricemia	11/155 (7.1%)	29/194 (14.9%)	0.0223

N—number of patients, overweight—defined as BMI < 30 kg/m^2^, obesity—defined as BMI ≥ 30 kg/m^2^, T2DM—type 2 diabetes mellitus, statistical test applied—Chi-Square test.

**Table 3:** Other comorbidities present in both groups.

	Group 1—TG/HDLc > 2.5 (n = 155)	Group 2—TG/HDLc < 2.5 (n = 194)	*p*-Value
n	155 (44.4%)	194 (55.6%)	
Hydrostatic varicose veins	30/155 (19.4%)	20/194 (10.3%)	0.0167
Chronic obstructive pulmonary disease	32/155 (20.6%)	36/194 (18.6%)	0.6250
Bronchial asthma	19/155 (12.3%)	15/194 (7.7%)	0.1571
Hypothyroidism	6/155 (3.9%)	12/194 (6.2%)	0.3320

n—number of patients, statistical test applied—Chi-Square test.

**Table 4:** Characteristics related to atrial fibrillation patients.

	Group 1—TG/HDLc > 2.5 (n = 155)	Group 2—TG/HDLc < 2.5 (n = 194)	*p*-Value
n	155 (44.4%)	194 (55.6%)	
Oral anticoagulation therapy	61/155 (39.4%)	77/194 (39.7%)	0.9492
Multiple hospital admissions	37/155 (23.9%)	30/194 (15.5%)	0.0479
Hospitalized for atrial fibrillation	72/155 (46.5%)	85/194 (43.8%)	0.6232
Anxiety–depressive disorder	25/155 (16.1%)	34/194 (17.5%)	0.7298
Depressive syndrome	19/155 (12.3%)	17/194 (8.8%)	0.2943

n—number of patients, statistical test applied—Chi-Square test.

**Table 5:** Cardiovascular comorbidities present in both groups.

	Group 1—TG/HDLc >2.5 (n = 155)	Group 2—TG/HDLc < 2.5 (n = 194)	*p*-Value
n	155 (44.4%)	194 (55.6%)	
HF	116/155 (74.8%)	140/194 (72.2%)	0.5751
HFpEF	37/155 (23.9%)	50/194 (25.8%)	0.6836
HFrEF	79/155 (51.0%)	90/194 (46.4%)	0.3960
HTN	132/155 (85.2%)	168/194 (86.6%)	0.7015
CCS	100/155 (64.5%)	104/194 (53.6%)	0.0402
Carotidian Atheromatosis	36/155 (23.2%)	37/194 (19.1%)	0.3438

n—number of patients, HF—heart failure, HFpEF—heart failure with preserved ejection fraction, HFrEF—heart failure with reduced ejection fraction, HTN—hypertension, CCS—chronic coronary syndrome, statistical test applied—Chi-Square test.

**Table 6:** Presence of cerebrovascular and neurological conditions in the two groups.

	Group 1—TG/HDLc > 2.5 (n = 155)	Group 2—TG/HDLc < 2.5 (n = 194)	*p*-Value
n	155 (44.4%)	194 (55.6%)	
Ischemic Stroke	58/155 (37.4%)	41/194 (21.1%)	0.0008
Cortical Atrophy	18/155 (11.6%)	4/194 (2.1%)	0.0003
Cognitive Decline	12/155 (7.7%)	9/194 (4.6%)	0.2265
Cerebral Lacunarism	15/155 (9.7%)	8/194 (4.1%)	0.0380
Parkinson’s Disease	9/155 (5.8%)	2/194 (1.0%)	0.0113

n—number of patients, statistical test applied—Chi-Square test.

**Table 7:** Cardiovascular and metabolically active medications used by patients in both cohorts.

	Group 1—TG/HDLc > 2.5 (n = 155)	Group 2—TG/HDLc < 2.5 (n = 194)	*p*-Value
N	155 (44.4%)	194 (55.6%)	
Vitamin K antagonist anticoagulant	123/155 (79.4%)	153/194 (78.9%)	0.7226
Beta-blocker	65/155 (41.9%)	66/194 (34.0%)	0.9113
ACEI	59/155 (38.1%)	53/194 (27.3%)	0.1298
Angiotensin receptor blockers	101/155 (65.2%)	127/194 (65.5%)	0.8822
Spironolactone	99/155 (63.9%)	103/194 (53.1%)	0.0329
Furosemide	17/155 (11.0%)	26/194 (13.4%)	0.0430
Amiodarone	7/155 (4.5%)	20/194 (10.3%)	0.7035
Propafenone	10/155 (6.5%)	11/194 (5.7%)	0.0444
Statin	59/155 (38.1%)	70/194 (36.1%)	0.9530
Allopurinol	61/155 (39.4%)	98/194 (50.5%)	0.7607
Metformin	12/155 (7.7%)	13/194 (6.7%)	0.1292
Alprazolam	123/155 (79.4%)	153/194 (78.9%)	0.7083
PPI	23/155 (14.8%)	8/194 (4.1%)	0.0403

N—Number of patients, PPI—Proton pump inhibitor, ACEI—Angiotensin-converting enzyme inhibitor, Test performed—Chi-Square test.

**Table 8:** Comparative analysis of age, glomerular filtration rate, and the main assessed electrolytes.

Characteristics	Group 1—TG/HDLc > 2.5 Mean	SD	95% CI	Group 2—TG/HDLc < 2.5 Mean	SD	95% CI	*p*-Value	Difference
Age	70.8387	10.6145	69.1544 to 72.5230	67.6443	11.1872	66.0602 to 69.2285	0.0067	−3.19
eGFR	59.1272	21.8694	55.6571 to 62.5974	59.4189	23.2052	56.1329 to 62.7048	0.9042	0.29
Na	140.1548	4.2474	139.4809 to 140.8288	140.0515	3.8235	139.5101 to 140.5930	0.8137	0.024
K	4.1587	0.5846	4.0659 to 4.2515	4.1830	0.6581	4.0898 to 4.2762	0.7157	0.38

eGFR—estimated glomerular filtration rate, calculated using KADOQI automated formula, Na—sodium, K—potassium.

**Table 9:** Comparative analysis of ischemic stroke count and the risk of thrombotic events and bleeding.

Characteristics	Group 1—TG/HDLc > 2.5 Mean	SD	95% CI	Group 2—TG/HDLc < 2.5 Mean	SD	95% CI	*p*-Value	Difference
Ischemic strokes numbers	0.4581	0.7318	0.3419 to 0.5742	0.2526	0.5875	0.1694 to 0.3358	0.0048	0.23
HAS-BLED	3.9419	1.1965	3.7521 to 4.1318	3.8402	1.2132	3.6684 to 4.0120	0.4334	0.12
CHA_2_DS_2_-VA	4.6194	1.7738	4.3379 to 4.9008	4.3711	1.8370	4.1110 to 4.6313	0.2037	−0.24

HAS-BLED: H—Hypertension; A—Abnormal renal and liver function; S—Ischemic Stroke; B—Bleeding; L—Labile INRs; E—Elderly Age> 65 ani; D—Drugs or alcohol CHA_2_DS_2_-VA; C—Congestive heart failure; H—Hypertension; A—Age; D—Diabetes mellitus; S—Ischemic Stroke; V—Vascular disease.

**Table 10:** Main laboratory tests.

Characteristics	Group 1—TG/HDLc > 2.5 Mean	SD	95% CI	Group 2—TG/HDLc < 2.5 Mean	SD	95% CI	*p*-Value	Difference
TG/HDLc	5.7239	6.1779	4.8491 to 6.5987	1.5829	0.5240	1.4998 to 1.6661	<0.0001	4.14
TGs	166.4175	85.3700	154.3287 to 178.5064	80.4452	22.6097	76.8576 to 84.0327	<0.0001	85.97
HDLc	34.8763	11.0108	33.3171 to 36.4355	53.7935	14.5346	51.4873 to 56.0998	<0.0001	−18.91
LDLc	110.6340	44.3461	104.3544 to 116.9137	90.4065	34.2005	84.9797 to 95.8332	<0.0001	20.22
Total Cholesterol	172.8505	51.0352	165.6237 to 180.0774	154.6065	40.5904	148.1658 to 161.0471	0.0003	18.24
Uric Acid	6.0701	3.1577	5.6230 to 6.5172	5.2981	2.9091	4.8365 to 5.7597	0.0183	0.77
Glycaemia	151.3041	82.1478	139.6716 to 162.9367	135.4581	51.1228	127.3462 to 143.5700	0.0281	15.84

TG/HDLc: TG-/HDLc cholesterol ratio; LDLc: Low-density lipoprotein; HDLc: High-density lipoprotein; Test performed: Welch’s *t*-test and unpaired student’s *t*-test.

**Table 11:** Odds ratio and risk ratio analysis for comorbidities present in Groups 1 and 2.

Group 1	ODDS RATIO	*p*-Value	95% C.I.	RR	*p*-Value	95% C.I.
Obesity	1.6656	0.0362	1.0334 to 2.6846	1.4426	0.0391	1.0184 to 2.0434
Multiple admissions	1.7141	0.0490	1.0024 to 2.9312	1.5437	0.0493	1.0014 to 2.3796
Hydrostatic varicose veins	2.0880	0.0181	1.1337 to 3.8455	1.8774	0.0187	1.1107 to 3.1735
Diabetes mellitus	2.2380	0.0007	1.4030 to 3.5702	1.7275	0.0011	1.2450 to 2.3969
Metabolic syndrome	1.8989	0.0131	1.1443 to 3.1510	1.6255	0.0149	1.0993 to 2.4036
Dyslipidemia	1.6716	0.0327	1.0432 to 2.6786	1.1558	0.0369	1.0088 to 1.3242
Ischemic stroke	2.2313	0.0009	1.3891 to 3.5843	1.7706	0.0010	1.2607 to 2.4866
Cortical atrophy	1.1421	0.0045	1.1432 to 2.3487.	1.3267	0.0056	1.0787 to 1.8734
Cerebral lacunarism	6.2409	0.0012	2.0661 to 18.8516	5.6323	0.0014	1.9462 to 16.3000
Parkinson’s disease	2.4911	0.0434	1.0274 to 6.0398	2.3468	0.0444	1.0216 to 5.3910
Ischemic heart disease	1.0374	0.0404	1.01 to 1.05	1.09	0.0385	1.05 to 1.12
Hyperuricemia	2.3008	0.0251	1.1097 to 4.7704	2.1064	0.0272	1.0874 to 4.0801
**Group 2**	**ODDS** **RATIO**	***p*** **-Value**	**95% C.I.**	**RR**	***p-*** **Value**	**95% C.I.**
Obesity	0.6004	0.0362	0.3725 to 0.9677	0.6932	0.0391	0.4894 to 0.9819
Multiple admissions	0.5834	0.0490	0.3412 to 0.9976	0.6478	0.0493	0.4202 to 0.9986
Hydrostatic varicose veins	0.4789	0.0181	0.2600 to 0.8820	0.5326	0.0187	0.3151 to 0.9003
Diabetes mellitus	0.4468	0.0007	0.2801 to 0.7128	0.5789	0.0011	0.4172 to 0.8032
Metabolic syndrome	0.5266	0.0131	0.3174 to 0.8739	0.6152	0.0149	0.4160 to 0.9097
Dyslipidemia	0.5982	0.0327	0.3733 to 0.9586	0.8652	0.0369	0.7552 to 0.9912
Ischemic stroke	0.4482	0.0009	0.2790 to 0.7199	0.5648	0.0010	0.4022 to 0.7932
Cortical atrophy	0.1602	0.0012	0.05305 to 0.4840	0.1775	0.0014	0.06135 to 0.5138
Cerebral lacunarism	0.4014	0.0434	0.1656 to 0.9733	0.4261	0.0444	0.1855 to 0.9789
Parkinson’s disease	0.90	0.0385	0.55 to 0.93	0.96	0.0404	0.79 to 0.98
Ischemic heart disease	0.71	0.0251	0.34 to 0.78	0.74	0.0251	0.39 to 0.89

RR—risk ratio; C.I.—confidence interval; Test—Chi-Square test; Metabolic syndrome—metabolic syndrome; Diabetes mellitus—diabetes mellitus.

**Table 12:** Association of the TG/HDLc ratio with other metabolic parameters.

	Correlation Coefficient r	*p*-Value	95% C.I. for r
TG/HDLc vs. glucose	0.17211	0.0013	0.06736 to 0.2712
TG/HDLc vs. LDLc	0.05717	0.2869	−0.04811 to 0.1612
TG/HDLc vs. uric acid	0.04065	0.4490	−0.06461 to 0.1450

C.I.—confidence interval; TG/HDLc—TG-/HDLc cholesterol ratio; LDLc—low-density lipoprotein cholesterol; HDLc—high-density lipoprotein cholesterol; Test performed: Pearson correlation.

**Table 13:** Secondary phenotype-clustering model: multivariable logistic regression for correlates of membership in Group 1 (TG/HDLc > 2.5).

Variable	β Coefficient	Std. Error	Wald χ^2^	*p*-Value	Odds Ratio (OR)	95% CI for OR
Ischemic stroke	0.57	0.26	4.97	0.0258	1.77	1.07–2.92
Cortical atrophy	1.50	0.59	6.55	0.0105	4.48	1.42–14.14
Cerebral lacunarism	0.37	0.53	0.50	0.4817	1.45	0.52–4.07
Parkinson’s disease	1.50	0.81	3.41	0.0648	4.48	0.91–22.03
Type 2 diabetes mellitus	1.51	0.24	38.49	<0.0001	4.53	2.81–7.29
Dyslipidemia	1.09	0.14	4.14	<0.0001	1.94	1.25–4.43
Hyperuricemia	1.34	0.37	12.99	0.0003	3.83	1.84–7.95
Constant	−0.53	0.13	15.96	0.0001		

β—logistic regression coefficient; CI—confidence interval.

## Data Availability

The original contributions presented in this study are included in the article material. Further inquiries can be directed to the corresponding authors.
